# Apoptosis induction in Jurkat cells and sCD95 levels in women's sera are related with the risk of developing cervical cancer

**DOI:** 10.1186/1471-2407-8-99

**Published:** 2008-04-11

**Authors:** Adriana Aguilar-Lemarroy, Jose E Romero-Ramos, Vicente Olimon-Andalon, Georgina Hernandez-Flores, Jose M Lerma-Diaz, Pablo C Ortiz-Lazareno, Gilberto Morgan-Villela, Susana del Toro-Arreola, Alejandro Bravo-Cuellar, Luis F Jave-Suarez

**Affiliations:** 1 División de Inmunología, Centro de Investigación Biomédica de Occidente, Instituto Mexicano del Seguro Social (IMSS), Guadalajara, Jalisco, México; 2 Hospital General de Zona No.9, IMSS, Ciudad Guzmán, Jalisco, México; 3 División de Oncología-Hematología, Centro Médico de Occidente, – IMSS, Guadalajara, Jalisco, México; 4 Laboratorio de Inmunología, Departamento de Fisiología, Centro Universitario de Ciencias de la Salud, Universidad de Guadalajara, Guadalajara, Jalisco, México

## Abstract

**Background:**

Currently, there is clear evidence that apoptosis plays an important role in the development and progression of tumors. One of the best characterized apoptosis triggering systems is the CD95/Fas/APO-1 pathway; previous reports have demonstrated high levels of soluble CD95 (sCD95) in serum of patients with some types of cancer. Cervical cancer is the second most common cancer among women worldwide. As a first step in an attempt to design a minimally invasive test to predict the risk of developing cervical cancer in patients with precancerous lesions, we used a simple assay based on the capacity of human serum to induce apoptosis in Jurkat cells. We evaluated the relationship between sCD95 levels and the ability to induce apoptosis in Jurkat cells in cervical cancer patients and controls.

**Methods:**

Jurkat cells were exposed to serum from 63 women (20 healthy volunteers, 21 with cervical intraepithelial neoplasia grade I [CIN 1] and 22 with cervical-uterine carcinoma). The apoptotic rate was measured by flow cytometry using Annexin-V-Fluos and Propidium Iodide as markers. Serum levels of sCD95 and soluble CD95 ligand (sCD95L) were measured by ELISA kits.

**Results:**

We found that serum from almost all healthy women induced apoptosis in Jurkat cells, while only fifty percent of the sera from women with CIN 1 induced cell death in Jurkat cells. Interestingly, only one serum sample from a patient with cervical-uterine cancer was able to induce apoptosis, the rest of the sera protected Jurkat cells from this killing. We were able to demonstrate that elimination of Jurkat cells was mediated by the CD95/Fas/Apo-1 apoptotic pathway. Furthermore, the serum levels of sCD95 measured by ELISA were significantly higher in women with cervical cancer.

**Conclusion:**

Our results demonstrate that there is a strong correlation between low levels of sCD95 in serum of normal women and higher apoptosis induction in Jurkat cells. We suggest that an analysis of the apoptotic rate induced by serum in Jurkat cells and the levels of sCD95 in serum could be helpful during the prognosis and treatment of women detected with precancerous lesions or cervical cancer.

## Background

A balance between apoptosis and cell proliferation are crucial features for the maintenance of homeostasis in multicellular organisms [1]. In malignant cells, apoptotic pathways are often disturbed, leading to uncontrollable growth and to resistance to anti-tumor treatment [2,3]. It is now well established that apoptosis plays an important role in the regulation of tumor progression [4,5]. Diverse molecular mechanisms, such as overexpression of anti-apoptotic proteins, inactivation of death receptors and mutations or epigenetic regulation of tumor suppressor genes, have been implicated in the failure of apoptosis in tumor cells [6-8].

Anti-apoptotic factors act directly by interfering with death receptor activation or indirectly by triggering an intracellular response that perturbs the apoptotic signaling cascades. One of the best characterized systems that triggers apoptosis is the CD95/Fas/APO-1 pathway [9-11]. CD95 is a member of the tumor necrosis factor receptor superfamily [12,13] that induces apoptosis in a variety of cell types. It is characterized by an intracellular domain, the "death domain". After CD95 ligand (CD95L) binding, the death domain attracts the intracellular adaptor protein FADD [14], which in turn recruits the "initiator" procaspase-8 and procaspase-10, forming a protein complex called DISC (death-inducing signaling complex) [15,16]. After autocatalytic activation of procaspase-8 at the DISC, active initiator caspase-8 can either directly or indirectly (via the mitochondrial pathway) activate downstream effector caspases (caspase-3, -6 and -7), leading to the cleavage of cellular proteins and subsequent apoptosis [9].

CD95 consists of two isoforms, one of them is anchored to the cellular membrane (mCD95) and the other one is present in a soluble form (sCD95). The first contains a single transmembrane region and induces apoptosis in normal or tumor cells, whereas the sCD95 lacks the transmembrane domain as a result of an alternative splicing and is thought to block apoptosis by CD95L binding [17]. Previous reports have demonstrated high levels of sCD95 in serum of patients with different malignancies such as bladder, breast, renal cell, hepatocellular and gynecological carcinomas [18-24].

CD95L is a 37 kDa membrane protein belonging to the TNF family, however, a soluble form is generated by a metalloproteinase-like protease and it is suggested that sCD95L prevents the recognition of tumor cells by binding to and inducing apoptosis in the cytotoxic T-cells [25]. It was reported that serum from healthy individuals does not contain detectable levels of sCD95L, whereas the presence of sCD95L has been noted in the serum of patients with some types of neoplasias [26-28].

Cervical cancer is the second most common cancer among women worldwide and represents the first cause of cancer death in developing countries, with an estimated of 493,000 new cases and 274,000 deaths during 2002 [29]. Infection with high-risk human papilloma virus (HPV) is considered the major etiological factor of premalignant lesions and cervical cancer [30,31]. Virtually almost 100% of cervical carcinoma samples have been shown to be positive for the presence of HPV-DNA [32]. The screening for cervical cancer and its precursor lesions currently employs the Pap smear, but this test is subjective and has relatively low sensitivity. The combination of the Pap test with HPV molecular detection achieves significant improvements in sensitivity for the detection of cervical cancer, but the last technique is not routinely employed due to methodological and economical reasons. Alternatively, the use of p16INK4a has been proposed as a prognostic marker for progression [33,34]; however, disadvantages of this method are that it is mainly confined to biopsies and it is also subjective depending on the pathologist's experience. At present in developing countries, women with cervical intraepithelial neoplasia grade 1 are normally maintained under observation, since almost 60% of these cases revert spontaneously [35,36]. To predict which of those patients are at a higher risk for progression to cervical cancer, it is necessary to look for new simple and low cost complementary prognostic methods. In the current study we used a very simple method based on the capacity of human serum to induce apoptosis in Jurkat cells in an effort to identify a prognostic marker or method which could assist in determining or predicting the risk of developing cervical cancer.

## Methods

### Patients

The study group consisted of 22 women with clinical and histopathological diagnosis of squamous cell carcinoma of the cervix, 21 women with CIN 1 (Cervical Intraepithelial Neoplasia grade 1 – mild dysplasia) and 20 healthy female volunteers. The age of cancer patients, CIN 1, and control group ranged from 30 to 83, 22 to 55, and 22 to 46 years, respectively. It is important to mention that patients included in this study did not receive any prior treatment (chemotherapy, radiotherapy or surgery). All patients signed an informed consent form approved by the Ethical Committee of the Instituto Mexicano del Seguro Social.

### Serum samples

Sera from untreated patients with cervical intraepithelial neoplasias grade 1 and with cervical cancer were obtained at Centro Médico Nacional de Occidente – IMSS. Control serum samples were obtained from healthy donors. All serum samples were obtained from peripheral blood by venipuncture after centrifugation at 2000 rpm for 15 minutes, aliquoted and stored at -70°C until used.

### Cell Culture

JURKAT and JURKAT^R ^(kindly obtained from Dr. Peter Krammer, DKFZ-Heidelberg, Germany) were cultured routinely in RPMI 1640 medium, supplemented with 10% fetal calf serum, 100 U/mL penicillin and 100 μg/mL streptomycin. All products mentioned before were obtained from GIBCO™ Invitrogen Corporation. Cultures were maintained at 37°C in a humidified atmosphere with 5% CO_2_. JURKAT^R ^is a JURKAT variant resistant to CD95-mediated apoptosis obtained after continuous exposure to agonistic Apo-1 antibody [37].

### Exposure of sera to Jurkat cells and apoptosis detection

To test the rate of apoptosis induced by sera from the different groups, JURKAT or JURKAT^R ^cells were seeded at a density of 2.5 × 10^5 ^cells per well in 1 mL RPMI medium in 6-well plates. Afterwards, 0.5 mL of serum from the different women was added. After 3 days of incubation, cell death was measured by flow cytometry using propidium iodide (Cat. P4864, Sigma-Aldrich, Germany) and Annexin-V-Fluos (Cat. 1828681, Roche Applied Science, Germany) as recommended by the manufacturers. For each sample, 10,000 events were analyzed in an Epics XL – MCL™ Flow Cytometer (Beckman Coulter, USA) using the FL-1 and FL-3 detector filters. Each serum was tested 3 to 5 times in independent experiments.

### Induction of apoptosis by CD95L

To corroborate the sensitivity of JURKAT and JURKAT^R ^cells to CD95L-induced apoptosis, 3.5 × 10^5 ^cells were seeded in 6-well plates and exposed to 5 μg/mL anti-Fas-human, activating- clone CH11 (Cat. 05-201, Lot. 33574, Upstate-Millipore Corporation), in a final volume of 1 mL RPMI medium. After 24 hours of incubation, apoptosis was measured by flow cytometry using Annexin-V-Fluos and Propidium Iodide as markers. Flow cytometry was performed in a FACSAria cytometer using for acquisition and analysis the FACSDiva software (Becton Dickinson, USA).

### Detection of sCD95 and sCD95L in serum

Serum concentrations of sCD95 and sCD95L were measured using the Human APO-1/FAS ELISA Kit (Cat. KHS9502, BioSource International) and the Human sFAS Ligand ELISA Kit (Cat. KHS9521, BioSource International), according to the manufacturer's specifications. The ranges of the kits for sCD95 and sCD95L were 0.23–15 ng/mL and 0.72–12.92 ng/mL, respectively. The sensitivity for APO-1/FAS is <20 pg/mL and for sFAS Ligand 0.1 ng/mL.

### Statistical analysis

Differences observed in the sensitivity of Jurkat cells to undergo apoptosis or in the sCD95 levels detected in sera between healthy controls, CIN 1 and cancer patients were analyzed using paired samples T-tests. To evaluate the correlation between apoptosis and sCD95 levels, Spearman's correlation coefficient was calculated. Results were considered statistically significant when *p *value was less than 0.05 and are presented as mean ± standard deviation. Analyses of all data were performed with the SPSS software version 12.0 (Chicago, Illinois, USA).

## Results

### Sera from patients with cervical cancer induce a cytoprotective effect that prevents apoptosis in Jurkat cells

**Figure 1:**
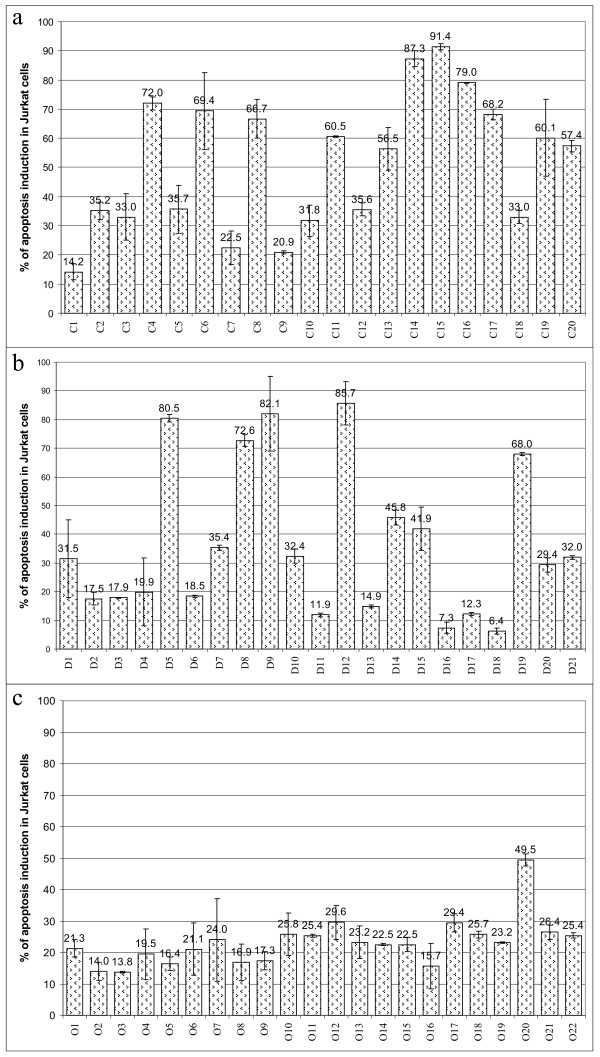
**Induction of apoptosis in Jurkat cells by women's sera**. The percentage of apoptosis induction in Jurkat cells was determined after three days exposure to sera from healthy controls (a), cervical intraepithelial neoplasia grade 1 patients (b) or cervical cancer patients (c). Apoptosis was measured by flow cytometry with Annexin-V-Fluos as a marker for 10,000 events. The graphic represents the results obtained from 3 to 5 independent experiments with standard deviation.

There is still controversy about the effect exerted by serum of cancer patients on T-lymphocytes. While there is evidence of T-lymphocyte apoptosis mediated by molecules contained in serum released by tumor cells [38], the opposite effect, that is, protection from apoptosis when T-lymphocytes were exposed to serum from cancer patients, was also reported [39]. To shed light on this discrepancy in the context of cervical cancer, we analyzed whether sera from patients with precancerous lesions or cervical cancer are able to protect or induce apoptosis in T-cells. For this purpose, we used Jurkat cells, a T-lymphocyte-derived cell line, as an apoptosis-sensitive model. This cell line is able to undergo apoptosis by triggering of the intrinsic and the extrinsic apoptosis pathways. Jurkat cells were exposed to serum from patients and healthy women volunteers. After three days of incubation, the apoptotic rates were measured. Our cohort was divided into three groups: control healthy women (Fig. 1a), women with CIN 1 (cervical intraepithelial neoplasia grade 1) (Fig. 1b), and women with cervical cancer (Fig. 1c). It is important to mention that serum from all patients included in this study was collected before any kind of treatment. As can be seen in Fig. 1a, sera obtained from healthy women were able to induce apoptosis in Jurkat cells in the majority of the cases (the percentages of apoptosis induction ranged from 14.2 to 91.4). Of the 20 analyzed sera, 17 (85%) were able to induce apoptosis in more than 30% of Jurkat cells. Using the same methodology, 11 out of 21 (52.4%) sera from women with precancerous lesions were able to induce apoptosis in more than 30% of Jurkat cells (Fig. 1b). Interestingly, when we tested sera from women with cervical cancer (Fig. 1c), only one out of 22 (4.5%) sera analyzed was able to induce apoptosis in more than 30% of Jurkat cells. It is important to emphasize that Jurkat cells grown only in RPMI culture medium were always included in duplicate as controls in each assay; Jurkat cell death obtained under these conditions ranged from 3 to 13% (data not shown). In summary, we observed that sera from healthy women tend to induce a high rate of apoptosis in Jurkat cells, whereas sera from patients with cervical cancer did not induce this killing. Additionally, approximately 50% of the sera obtained from precancerous lesions showed, in common with the samples from cancer patients, a failure to induce apoptosis. This suggests that these patients might be at a higher risk for the progression to malignant lesions.

### Serum from healthy women induced apoptosis in Jurkat cells through the CD95/Fas/Apo-1 pathway

**Figure 2:**
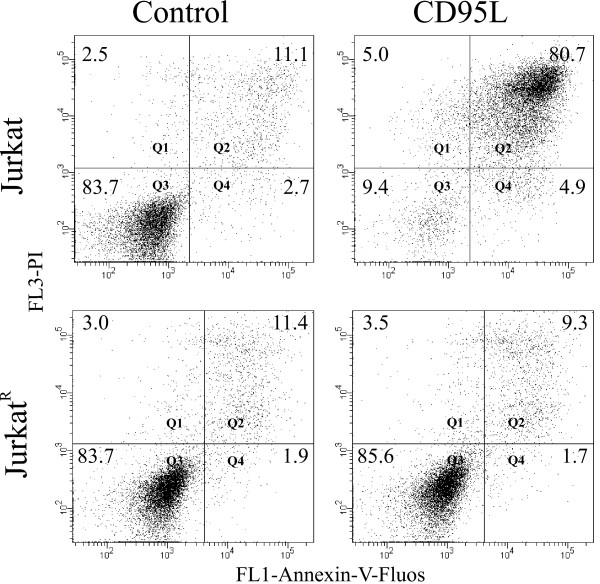
**Sensitivity of parental Jurkat and Jurkat^R ^cells to CD95L-induced apoptosis**. Jurkat and Jurkat^R ^cells were incubated 24 hours with CD95L as described in Methods, and the rate of apoptosis was measured by flow cytometry using Annexin-V-Fluos (x axis) and Propidium Iodide (y axis). Q3 represents living cells and Q2 represents cells undergoing apoptosis. The percentages of cells in each quadrant are given. The analysis was performed with the BD FACSDiva software. Control cells are untreated cells.

**Figure 3:**
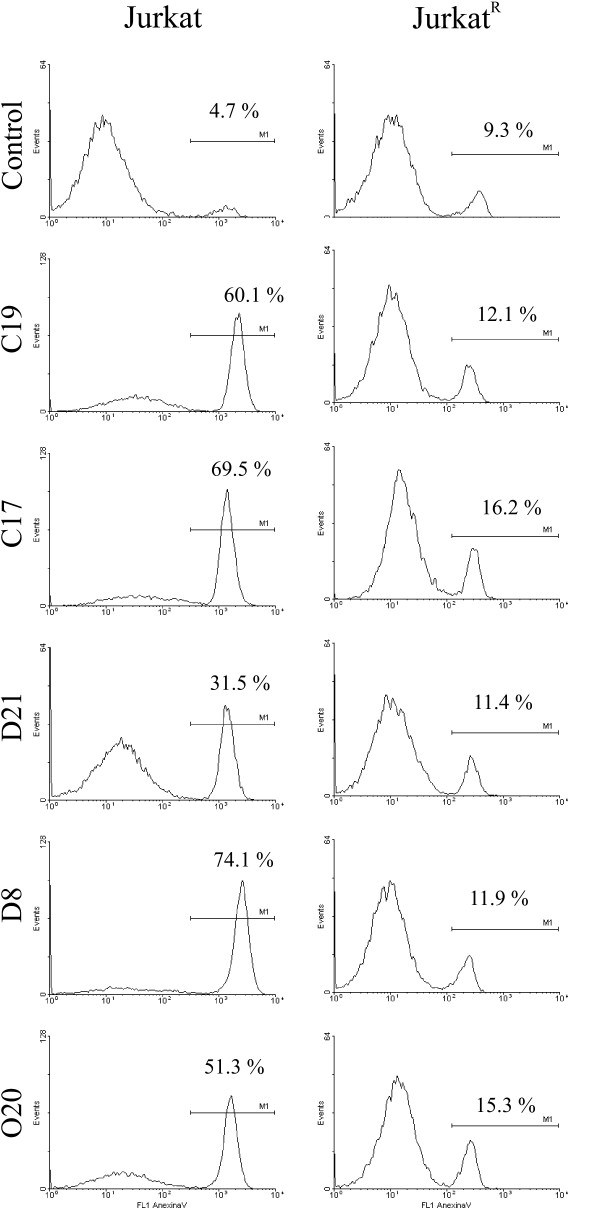
**Elimination of Jurkat cells is dependent on the CD95 pathway**. Jurkat and Jurkat^R ^cells were cultured in the presence of different sera obtained from control volunteers (C17, C19), women with CIN 1 (D8, D21) and serum from a woman with cervical cancer (O20). After 3 days of incubation at 37°C, the apoptosis induced in each T-cell line was measured by flow cytometry with Annexin-V-Fluos as a marker. Control: Jurkat or Jurkat^R ^cells grown only with complete RPMI medium. M1 represents the percentage of cells undergoing apoptosis. A total of 10,000 events were measured; analyses were performed using the WinMDI2.8 software. Histograms are representative of separate experiments performed at least twice per condition.

As previously mentioned, we used Jurkat cells to measure apoptosis induction by sera. These cells are responsive to a variety of apoptotic signals including death receptors and intrinsic signals. Questions arising from our previous experiments led us to attempt to elucidate the apoptotic pathway involved in the killing effect induced by healthy women's sera. To test the relevance of the CD95 pathway in this apoptotic process, we used a Jurkat derived CD95 resistant cell line (Jurkat^R^) [37]. First, we corroborated the CD95 sensitivity of Jurkat and Jurkat^R ^cells by incubating both cell lines with CD95 ligand (CD95L) and measuring the apoptotic rate after 24 hours by flow cytometry using Annexin-V-Fluos and Propidium Iodide (PI) as markers. As can be seen in Fig 2, parental Jurkat cells were highly sensitive to CD95L exposure (90.6% cell death). As expected, Jurkat^R ^cells were resistant to CD95L-induced apoptosis, despite the high concentration of CD95L used and the long time of incubation. After corroborating the apoptosis sensitivity, we used this resistant property of Jurkat^R ^cells to investigate the role of the CD95 pathway in the Jurkat killing induced by sera exposure. We selected those sera that previously demonstrated an ability to induce apoptosis in Jurkat cells and incubated them in parallel with both, Jurkat and Jurkat^R ^cells. After 3 days of incubation, apoptosis was measured by flow cytometry using Annexin and PI as previously mentioned. As depicted in Fig. 3, sera that induced apoptosis in Jurkat cells were unable to do so in Jurkat^R ^cells. The apoptotic rate induced in Jurkat cells ranged from 31.5% to 74.1%, while apoptosis induced in Jurkat^R ^cells was similar to that observed in control cells grown without serum exposure. These results strongly suggest an involvement of the CD95 pathway in the killing of Jurkat cells by sera from healthy women.

### sCD95 levels in serum of women with cervical cancer correlate with the inhibition of apoptosis observed in Jurkat cells

**Figure 4:**
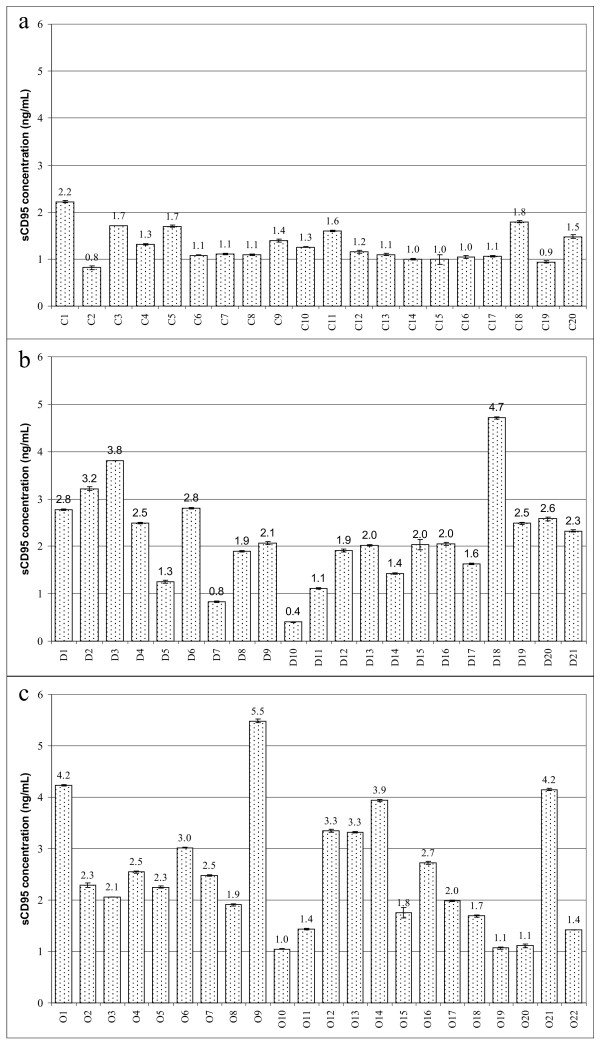
**sCD95 levels in serum**. Concentrations of soluble CD95 were measured by ELISA assays in sera obtained from healthy controls (a), CIN 1 patients (b), or cervical cancer patients (c). sCD95 expression is shown as ng/mL. The results are expressed as average ± SD of three measurements from 2 independent experiments.

Since it was shown that the CD95 pathway was involved in the elimination of Jurkat cells, we next analyzed the soluble CD95 receptor (sCD95) levels in serum. In order to perform this goal, we took advantage of commercially available kits for quantification of sCD95 protein levels and analyzed all sera included in this study. sCD95 levels found in healthy women's sera ranged mostly from 1.0 to 1.8 ng/mL; only one serum sample showed a sCD95 protein level over 2 ng/mL (Fig. 4a). Nevertheless, serum from patients with cervical cancer showed higher levels of sCD95 protein (Fig. 4c), in which the majority ranged between 2.0 and 4.2 ng/mL and one case showed 5.5 ng/mL. On the other hand, sera from the CIN 1 group showed in six cases a variation around 0.4 to 1.6 ng/mL of sCD95 levels, but the majority was over 1.9 ng/mL (Fig. 4b).

**Figure 5:**
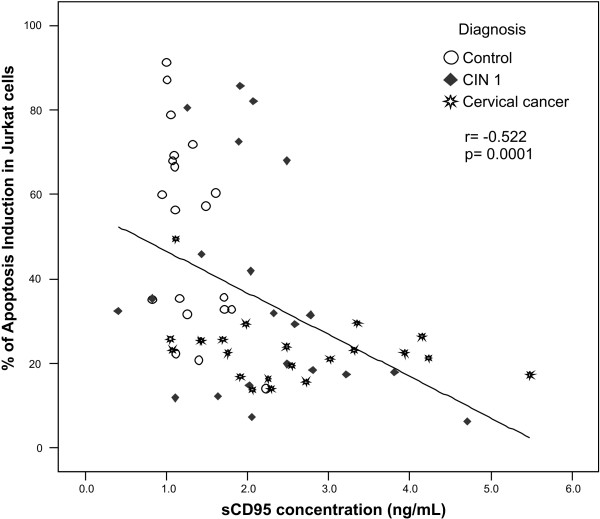
**Correlation between apoptosis induction in Jurkat cells and serum levels of sCD95**. The Spearman's correlation coefficient was calculated using the SPSS software; a significant but inverted correlation was observed (*p *= 0.0001, r = -0.522).

Statistical analysis of the data demonstrated that there is a strong correlation between high levels of sCD95 in serum and low apoptosis induction in Jurkat cells, as depicted in Fig. 5, where the correlation between apoptosis and sCD95 levels was calculated using the Spearman's correlation test. These results confirm the premise that sCD95 present in serum exerts a protective effect on Jurkat cells avoiding apoptosis (compare Fig. 1 with Fig. 4).

### The rate of apoptosis induction in Jurkat cells and the sCD95 levels in serum are statistically significant between healthy women and women with cervical cancer

**Figure 6:**
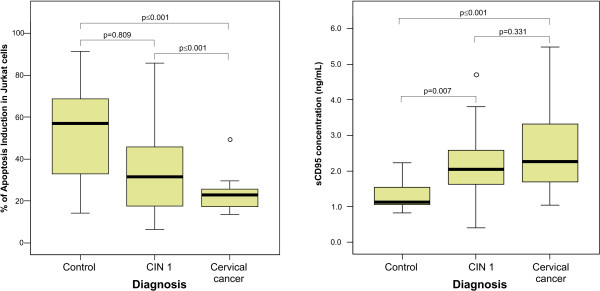
**Statistical analysis of apoptosis induction in Jurkat cells and serum levels of sCD95**. Box plot graphics show the correlation between the percentage of apoptosis induction in Jurkat cells and sCD95 expression levels in sera of the different groups: CONTROL (healthy volunteers), CIN 1 (women with cervical intraepithelial neoplasia grade 1) and CERVICAL CANCER. Graphics display the median (dark lines), 25^th ^percentile to the 75^th ^percentile (boxes), interquartile ranges (whiskers) and outliers (empty small circles) from our data. The statistical significance (*p *value) is given between the groups.

We performed a statistical analysis with our experimental data employing the SPSS software. T-Test and Levene's Test for equality of variances were achieved to compare the percentage of apoptosis induction and the sCD95 levels between different groups. Figure 6 displays in box plot graphics, the median, interquartile ranges and outliers from our data. As we can observe, there is a statistical significance between data obtained in the control group compared with data from the cervical cancer group. The significance was not only in the percentage of apoptosis induction, but also in the sCD95 levels. However, when we compared the data obtained from CIN 1 group with the data of control or cancer groups, we observed a statistical significance only in the percentage of apoptosis induction between CIN 1 and cancer and in the sCD95 expression between control and CIN 1. These results suggest that the percentage of apoptosis induction in Jurkat cells and the sCD95 levels present in serum could be important diagnostic and prognostic tools for precancerous lesions advancing to cervical cancer.

## Discussion

Currently, there is clear evidence that apoptosis plays an important role in the development and progression of tumors [5,40,41]. Apoptosis is regulated by a variety of extrinsic and intrinsic signals; one of the best known apoptosis-inducing pathways is the CD95 receptor-ligand system [41]. It has been shown that neoplastic cells have different alterations in cell death-involved proteins; some of which result in an impairment in the function or expression of CD95 and its ligand, which have been implicated in the pathogenesis of malignant diseases [10,41]. One of these alterations is the increased expression level of sCD95 found in the serum of patients with some types of cancer. The biological function of sCD95 is to bind the soluble or membrane-anchored CD95L; this binding has been shown to protect cells from CD95-mediated apoptosis and represents one method of evading immuno surveillance [8,17,42].

Taking advantage of this knowledge, the objective of this study was to design a minimally invasive test which may serve to predict the risk of developing cervical cancer. Based on the apoptosis sensitivity of Jurkat cells and the sCD95 levels found in serum we were able to identify a possible test system which, in addition, is inexpensive and quite robust. This goal was reached, since we observed a significant correlation between the inhibition of the sera's capacity to induce apoptosis in Jurkat cells and their sCD95 levels with the stage of disease. Our data obtained from 63 women show that the majority of sera from healthy volunteers can efficiently induce apoptosis in Jurkat cells; however, cervical cancer patients' sera induce only a low percentage of apoptosis; in women with CIN 1 lesions, we observed a 50% tendency to efficiently kill Jurkat cells (Fig. 1). Interestingly, more than 50% of the CIN 1 lesions regress spontaneously without treatment [35,36]. This result is in agreement with the report of Vejda S., et al., in which they established that plasma from cancer patients mediates protection against apoptosis. In their work, they induced apoptosis in Jurkat cells by adding anti-CD95 antibody or staurosporine supplemented with plasma samples from prostate, lung and breast cancer patients or from healthy human individuals. They found that the apoptotic indices of Jurkat cells were significantly reduced when cells were supplemented with plasma derived from cancer patients; in contrast, supplementation of medium with plasma from healthy human individuals resulted in higher apoptotic indices [39].

An additional goal in our study was to determine whether serum-induced apoptosis in Jurkat cells is specifically mediated by the CD95 pathway, this objective was met by using the CD95 resistant Jurkat^R ^cell line (Fig. 3); these cells were resistant to undergo apoptosis after incubation with different sera that were able to induce apoptosis in Jurkat cells. Since these results strongly suggested a pivotal role of CD95 in this process, we decided to measure its expression in the serum of all patients. Using ELISA assays, we found that sCD95 levels were elevated in the sera of women with cervical cancer when compared with those of healthy control women. Comparing our data with other malignancies, elevated levels of sCD95 in serum have been also observed in breast cancer [18,19], bladder [20] and renal carcinomas [21], advance melanoma [43], B-cell chronic lymphocytic leukemia (B-CLL) [44] and also in autoimmune rheumatic diseases [45]. Concerning gynecological tumors, it has been reported that serum levels of sCD95 are increased in women with uterine tumors in comparison with the control group [23]. In addition, Konno et al., examined the relationship between sCD95 levels and survival rate in patients with gynecological malignancies; they observed that patients diagnosed with cervical carcinoma have a better survival rate when they have serum sCD95 levels lowers than 1.5 ng/mL before therapy than those with a level higher than 1.5 ng/mL [24].

Despite the relevance of sCD95L levels found in serum in other kind of carcinomas [22,46-48], we determined that serum levels of sCD95L in women were not relevant as a biomarker in cervical cancer, since we found undetectable concentrations of this protein by ELISA assays in all women analyzed (data not shown). These data are contradictory to the observations reported by Kondera-Anasz, et al., in which they reported a significant increase of sCD95L in serum of women with uterine tumor compared to the control group [23]; however, one explanation to this discrepancy could be the use of different commercially available ELISA kits for the sCD95L detection. Concerning healthy volunteers, our data are in agreement with reports that have found very low levels of sCD95L in serum of such controls [23,49].

Looking at the sum of our data, we conclude that the percentage of apoptosis induction in Jurkat cells and the sCD95 levels present in serum of women could be important diagnostic and prognostic tools for cervical cancer, however, a follow-up study for at least 2 years must be made in order to confirm whether the cervical lesions found in women that are diagnosed as CIN 1, with high serum levels of sCD95 and a low capacity to eliminate Jurkat cells, progress readily to CIN 2. Additionally, it will be of interest to determine whether these observations are specific for cervical cancer or if they are found in other cancers as well.

## Conclusion

In conclusion, our results show an anti-apoptotic effect of sera derived from patients with cervical cancer. Additionally, we were able to show higher levels of soluble CD95 in serum of women with cancer than in those of healthy controls. We conclude that the disparate levels observed in patients with cervical cancer versus control patients protect Jurkat cells from apoptosis and could be an important mechanism for evading the antitumor immune response. We suggest that an analysis of the apoptotic rate induced by serum in Jurkat cells and the levels of soluble CD95 in serum could be helpful during the prognosis and treatment of women detected with precancerous lesions or cervical cancer.

## Competing interests

The author(s) declare that they have no competing interests.

## Authors' contributions

AAL, JERR and VOA carried out the experimental work, GHF, STA and PCOL participated in the classification and characterization of patients, JMLD, GMV and ABC were involved in the recruitment of patients and controls. AAL and LFJS performed the statistical analysis, conceived and designed the study and wrote the manuscript. All authors helped to draft the manuscript and read and approved this final version.

## Pre-publication history

The pre-publication history for this paper can be accessed here:


